# Triphase Separation of a Ternary Symmetric Highly Viscous Mixture

**DOI:** 10.3390/e20120936

**Published:** 2018-12-06

**Authors:** Andrea Lamorgese, Roberto Mauri

**Affiliations:** 1 Departmento di Ingegneria dell’Energia, dei Sistemi, del Territorio e delle Costruzioni, Università of Pisa, Largo Lazzarino 1, 56122 Pisa, Italy; 2 Departmento di Ingegneria Civile e Industriale, Università of Pisa, Largo Lazzarino 1, 56122 Pisa, Italy

**Keywords:** spinodal decomposition, liquid–liquid phase transitions, diffusion, nonequilibrium thermodynamics

## Abstract

We discuss numerical results of diffusion-driven separation into three phases of a symmetric, three-component highly viscous liquid mixture after an instantaneous quench from the one-phase region into an unstable location within the tie triangle of its phase diagram. Our theoretical approach follows a diffuse-interface model of partially miscible ternary liquid mixtures that incorporates the one-parameter Margules correlation as a submodel for the enthalpic (so-called excess) component of the Gibbs energy of mixing, while its nonlocal part is represented based on a square-gradient (Cahn–Hilliard-type) modeling assumption. The governing equations for this phase-field ternary mixture model are simulated in 3D, showing the segregation kinetics in terms of basic segregation statistics, such as the integral scale of the pair-correlation function and the separation depth for each component. Based on the temporal evolution of the integral scales, phase separation takes place via the simultaneous growth of three phases up until a symmetry-breaking event after which one component continues to separate quickly, while phase separation for the other two seems to be delayed. However, inspection of the separation depths reveals that there can be no symmetry among the three components at any instant in time during a triphase segregation process.

## 1. Introduction

A ternary mixture of partially miscible liquids, which is homogeneous at some high temperature in the one-phase region of its phase diagram, may separate into three phases after rapid quenching to an unstable location within the spinodal range of its phase diagram [1,2]. Presented herein is a numerical study of isothermal spinodal decomposition of a very viscous ternary liquid mixture in the limit of zero fluidity coefficient (further addressed below), following a thermal quench from a stable state in the one-phase region to an unstable location within the tie triangle of its phase diagram. Although our attention in this work will be restricted to nonreactive (symmetric) ternary liquid mixtures, it should be acknowledged that the behavior of reactive (not necessarily symmetric) ternary mixtures has received considerable attention over the past two decades [3,4,5,6,7,8,9,10]. In fact, even though spinodal decomposition in a number of reaction-diffusion systems is usually triggered by a thermal or compositional quench, actually the necessary displacement from a stable state in the one-phase region into the spinodal range can also occur by reaction, e.g., polymerization of a monomer when the polymer being formed is incompatible with one or more of the other components in the mixture. Such reaction-induced spinodal decomposition is particularly relevant to the design of new materials, such as, e.g., monolithic porous polymer or carbon materials, and consequently has been studied extensively over the past two decades, mostly by experiment [11,12,13,14,15,16,17,18]. However, in what follows, we will not address reaction-induced spinodal decomposition in any detail, since the primary objective of this work is to elucidate whether or not the assumption of a perfectly symmetric ternary system implies some kind of statistical symmetry during the triphase segregation process after spinodal decomposition triggered by a thermal quench. We will also briefly discuss segregation patterns (i.e., isosurfaces of concentration as a function of time) in two cases wherein phase separation for one component in a ternary mixture turns out to be faster or slower than that for the remaining components. Note that, since we focus on a ternary system with a vanishing ratio of capillary to viscous forces, the results reported herein may be considered as pertaining to ternary alloy systems or polymer blends. In fact, as is well known, the mechanical properties and rheology of ternary polymer blends are greatly influenced by their phase morphology, and, consequently, the most important application of phase separation dynamics to date has been to predict those phase morphologies as a function of controlling factors such as viscosity of components, composition, interfacial interaction between phases, and processing parameters. For the sake of the present discussion, we note that, while in a binary blend there are only two basic morphologies (particulate and bicontinuous), a large number of phase morphologies have been documented in ternary polymer blends, and, consequently, a taxonomy of phase morphologies has been the subject of a number of previous works; in particular, Nauman and He [19] showed 23 morphologies of which 18 were actually observed in their 2D simulation study but also included morphological types which reflected the topological limitations of 2D space. Once again, we emphasize that phase morphologies in a ternary polymer system are beyond the scope of the present work since the focus of this paper is on the triphase segregation kinetics following spinodal decomposition of a very viscous and symmetric ternary liquid mixture. Finally, we also acknowledge that significant progress in understanding the kinetics of ternary phase-separating mixtures in the absence of chemical reactions has been reported in a number of previous works [1,20,21,22,23,24,25].

An outline of the remainder of this paper is as follows. Below, we first present a brief review of the governing equations for our phase-field formulation, emphasizing that the ternary mixture model of interest herein is supposed to describe a nonideal liquid mixture with perfectly symmetric pure species; consequently, our phase-field formulation has been based on the one-parameter Margules correlation since that is the simplest excess Gibbs energy submodel one can write for a nonideal liquid mixture. In contrast, in some cases, more complex excess Gibbs energy submodels are required for modeling nonideal mixtures having a strong asymmetry among the component species (e.g., when two or more components are partially immiscible). That was specifically the case with the benzene-acetonitrile-water mixture considered in our previous work [26], where the phase-field ternary mixture model presented herein was generalized to incorporate the nonrandom, two-liquid (NRTL) equation [27,28,29] as an excess Gibbs energy submodel. Finally, in Section 3, using different initial concentration field perturbations, we discuss simulation results of a symmetric ternary mixture in terms of the integral scale of the pair-correlation function and the separation depth for each component. Conclusions are then presented.

## 2. Model Description

Below, we provide a brief summary of the governing equations for a diffuse-interface description of partially miscible ternary liquid mixtures, given that a detailed derivation of those equations has been presented elsewhere [2,26,30]. Consider a regular ternary mixture, whose component liquids have the same molar density, ρ. This mixture can be modeled within a diffuse-interface description by assuming that its free energy is the sum of a thermodynamic part and a nonlocal contribution [26,30,31,32,33,34], i.e.,

(1) $$G=\rho RT\int_{V}\tilde{g}\;d^{3}r\;\mathrm{with}\;\tilde{g}=g\left(x_{1},x_{2}\right)-\frac{a^{2}}{2}\left(\nabla x_{1}\cdot \nabla x_{2}+\nabla x_{1}\cdot \nabla x_{3}+\nabla x_{2}\cdot \nabla x_{3}\right),$$

where *g* is the (dimensionless) thermodynamic (i.e., coarse-grained) bulk free energy density, *T* the absolute temperature, *V* the volume, *R* the universal gas constant, while *a* is a characteristic length. Note that, since Equation (1) represents a coarse-grained expression of the free energy, the characteristic length is in no way equal to the actual interfacial thickness (still *a* is representative, on the mesoscale, of the typical interface thickness at local equilibrium). Assuming that the mixture has zero excess volume of mixing and zero excess entropy of mixing, the simplest expression [28,29,35] for the thermodynamic free energy density, *g*, corresponding to a perfectly symmetric, partially miscible ternary mixture, is the sum of an entropic, ideal part, and an enthalpic (so-called excess) part, with

(2) $$g=g_{0}+x_{1}\ln x_{1}+x_{2}\ln x_{2}+x_{3}\ln x_{3}+\Psi \left(x_{1}x_{2}+x_{1}x_{3}+x_{2}x_{3}\right).$$

Here, $g_{0}$ is the free energy of each pure component, while $x_{i}$ (i=1,2,3) denotes the molar (and mass) fraction of the *i*th species, and Ψ is the Margules parameter corresponding to any one component pair [35]. [Obviously, since we are considering an ideally, perfectly symmetric ternary mixture, the Margules parameter for the (i,j) component pair must be equal to those for the remaining pairs. Note that, for strongly nonideal systems (particularly for partially immiscible components when there is no symmetry among the pure species), we have shown a derivation of the species balance equations that incorporates NRTL as an excess Gibbs energy submodel [26], in place of the standard one-parameter Margules correlation employed in Equation (2).] When the mixture finds itself in a state of nonequilibrium, it evolves according to the equations governing dynamic processes, expressing standard conservation principles [36]. Herein, we assume isothermal conditions and therefore ignore the energy balance equation; consequently, the governing equations reduce to a pair of (nonreactive) species balance equations, that are normally coupled to the Navier–Stokes equation (in addition to the zero divergence constraint on the mass-averaged velocity that strictly applies to an isopycnic, regular mixture). Nondimensionalizing each species conservation equation (as well as the Navier–Stokes equation) based upon a diffusive scaling [37,38,39,40,41] gives rise to the so-called fluidity coefficient [42]

(3) $$\alpha \equiv \frac{RTa^{2}}{M_{W}\nu D},$$

with $M_{W}$ and ν denoting the molecular mass and kinematic viscosity of each pure component, while *D* denotes the (same) binary diffusivity for all component pairs. This dimensionless group can be introduced as an inverse capillary number [43], while it can also be interpreted as a Peclet number [33,34,38,39,40,41], i.e., the ratio of convective to diffusive mass fluxes in the species balance equations. In fact, in previous works [33,34,44,45,46,47,48,49], we have noted that, in low-viscosity systems, α is usually of order $O(10^{3}÷10^{6})$, while highly viscous mixtures (e.g., polymer melts and alloys) correspond to a vanishing fluidity coefficient. In the latter case (which is the focus of the work reported herein), the diffuse-interface model describes a diffusive (or antidiffusive) separation process in the absence of flow, and the species balance equations assume the particularly simple form seen earlier [26,30]:

(4) $$\begin{array}{cc} \frac{\partial x_{1}}{\partial t} & =\nabla \cdot \left\{-x_{1}x_{2}\nabla {\tilde{\mu }}_{23}+x_{1}\left(1-x_{1}\right)\nabla {\tilde{\mu }}_{13}\right\}, \end{array}$$

(5) $$\begin{array}{cc} \frac{\partial x_{2}}{\partial t} & =\nabla \cdot \left\{-x_{1}x_{2}\nabla {\tilde{\mu }}_{13}+x_{2}\left(1-x_{2}\right)\nabla {\tilde{\mu }}_{23}\right\}. \end{array}$$

These equations have been scaled based on the characteristic length *a* and the diffusive time $a^{2}/D$. The generalized chemical potential differences in Equations (4) and (5) can be readily obtained from the Bakhuis relation [50], ${\tilde{\mu }}_{i3}=\frac{\delta \tilde{g}}{\delta x_{i}}$ (i=1,2). In particular, we find

(6) $$\begin{array}{cc} {\tilde{\mu }}_{13} & =\ln\frac{x_{1}}{x_{3}}+\Psi \left(x_{3}-x_{1}\right)-\frac{a^{2}}{2}\nabla^{2}\left(x_{1}-x_{3}\right), \end{array}$$

(7) $$\begin{array}{cc} {\tilde{\mu }}_{23} & =\ln\frac{x_{2}}{x_{3}}+\Psi \left(x_{3}-x_{2}\right)-\frac{a^{2}}{2}\nabla^{2}\left(x_{2}-x_{3}\right). \end{array}$$

These relations, together with the equalities ${\tilde{\mu }}_{ij}={\tilde{\mu }}_{ik}+{\tilde{\mu }}_{kj}$ and ${\tilde{\mu }}_{ij}=-{\tilde{\mu }}_{ji}$, define all chemical potential differences for a ternary system. Finally, it is worth reiterating that Equations (4) and (5) constitute a system of fourth-order equations, which represents a generalization (specifically, a ternary version of Model B in the taxonomy of Hohenberg and Halperin [51]) of the classical Cahn–Hilliard equation to describe phase separation in binary mixtures [33,34,37,38,39,41,52,53,54].

## 3. Results and Discussion

Numerical methods employed herein for integrating the species balance equations above in a periodic box are exactly the same as discussed in a previous work [26] and therefore will not be repeated below. In fact, due to our assumption of periodic boundaries, initially we attempted simulations based on a pseudospectral spatial discretization [30,38,39,40,41,49], in conjunction with an adaptive temporal scheme (which had been successfully employed in simulations of dissolution or growth of a multicomponent drop into the continuous phase of another liquid [26,30]). Subsequently, even though our pseudospectral code achieved a stable integration, we realized (based on numerical tests) that a pseudospectral discretization does not preserve the scalar boundedness of the concentration fields. Consequently, the temporal evolution of the separation depth for some component usually went out of bounds, since at some locations in the computational domain the mass fraction for that component became slightly larger than one, or less than zero (or both) for a significant fraction of simulation time. However, assuming the same explicit adaptive temporal scheme as in our pseudospectral code, we also found that issues of scalar boundedness could be circumvented by changing the spatial discretization from pseudospectral to finite volume. (Incidentally, we chose a standard finite volume discretization based on a cell-centered variable arrangement, and using central differencing for both interpolation and differentiation; consequently, our scheme is second-order accurate in space since it relies on a uniform Cartesian grid.) As to the time marching, we also noticed that a stable integration could be achieved using the standard fourth-order Runge–Kutta scheme. Finally, we chose this last temporal scheme (in conjunction with a standard finite-volume discretization) in our production runs for calculating the results presented below.

**Figure 1:**
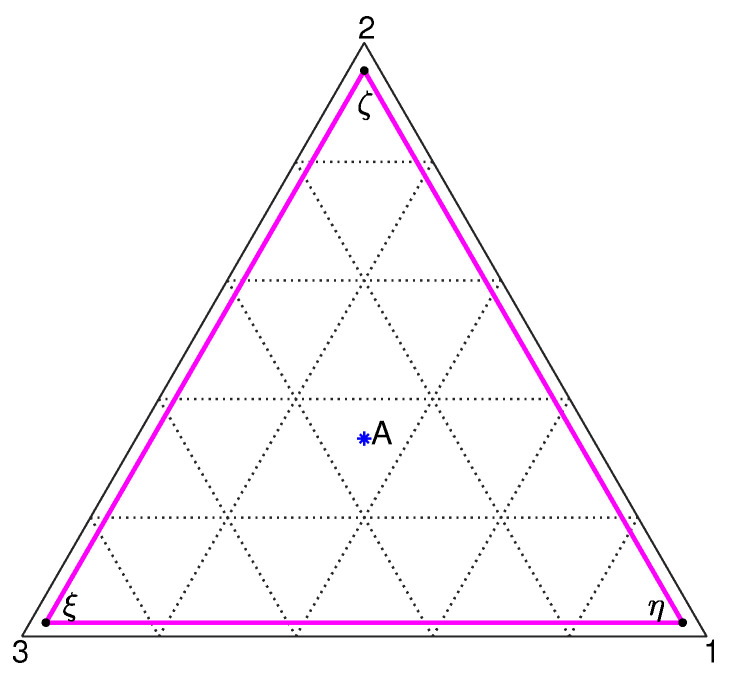
Phase diagram for an ideally perfectly symmetric ternary mixture with Ψ=4. Point A denotes an (unstable) equilibrium state [with $x_{A}=\left(\frac{1}{3},\frac{1}{3},\frac{1}{3}\right)$] corresponding to the initial mixture composition.

In our simulations we chose Ψ=4, since that was the value for the Margules parameter in the triphase separation simulation of an ideally perfectly symmetric ternary mixture reported by Park et al. [2]. In fact, for $\Psi =4>\frac{8}{3}$ the phase diagram for the mixture is a tie triangle [1], whose vertices represent the compositions of three coexisting phases (see Figure 1) at local equilibrium. If we let $x^{\xi }=\left(x,x,1-2x\right)$, $x^{\eta }=\left(1-2x,x,x\right)$, and $x^{\zeta }=\left(x,1-2x,x\right)$, the coordinates for the vertices are readily found by observing that all (thermodynamic) chemical potential differences reduce to either a trivial identity or the following relation

(8) $$\mu_{21}^{th}=\ln\frac{x}{1-2x}+\Psi \left(1-3x\right)=0\;⟹\;x^{*}=0.023.$$

Hence we find $x^{\xi }=\left(0.023,0.023,0.954\right)$, $x^{\eta }=\left(0.954,0.023,0.023\right)$, and $x^{\zeta }=\left(0.023,0.954,0.023\right)$.

**Figure 2:**
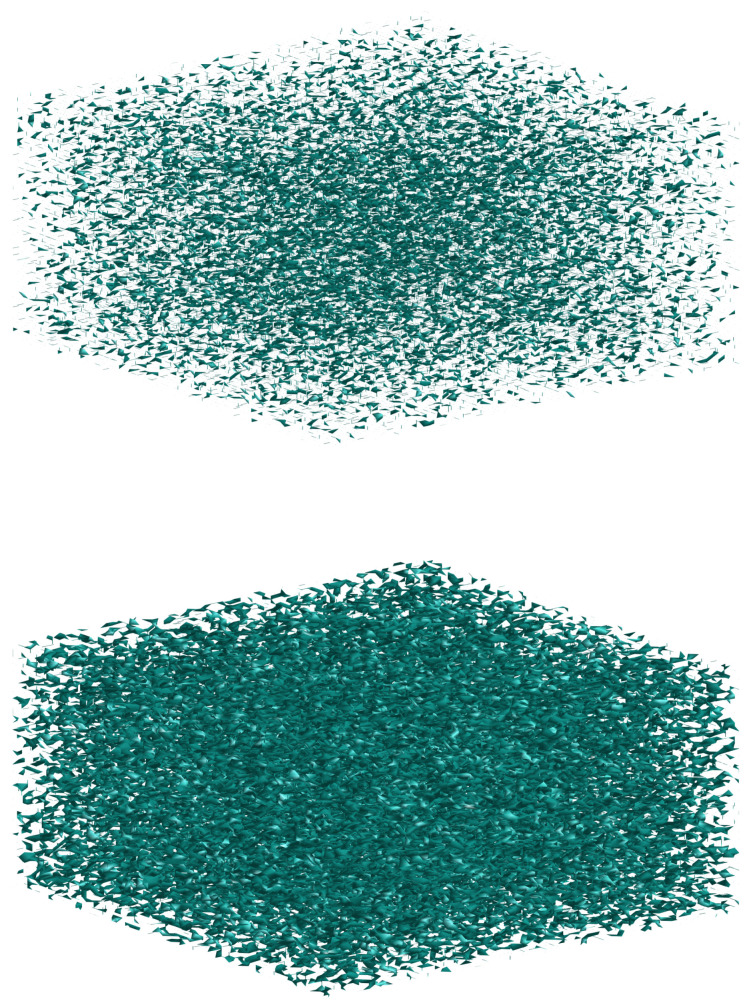
Isosurfaces of phase η ($x_{1}=0.95$, top) vs. those for phase ξ ($x_{3}=0.95$, bottom) at the same (non-dimensional) time $\tau =1.43\times 10^{-3}$ from 3D simulation (case I) of a ternary mixture with Ψ=4 on a $128^{3}$ grid.

**Figure 3:**
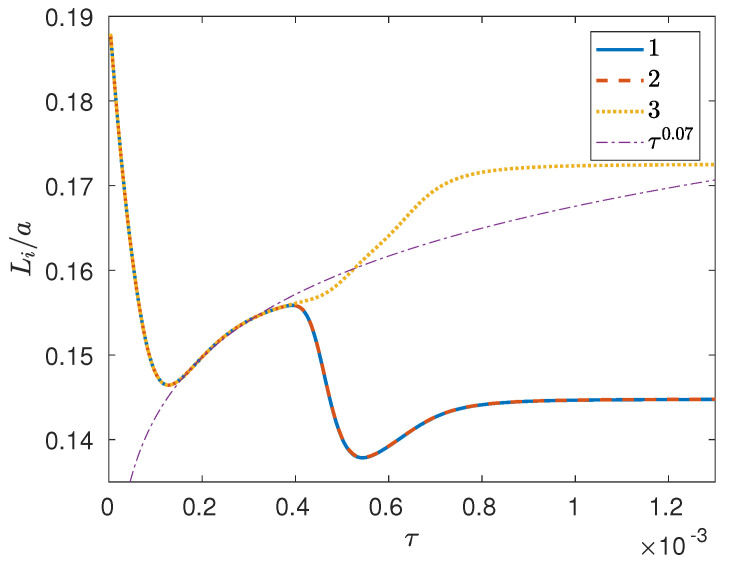
Integral scales vs. time (diffusive time units) from 3D phase separation simulation (case I) of a perfectly symmetric ternary mixture with Ψ=4 and global composition $x_{A}=\left(\frac{1}{3},\frac{1}{3},\frac{1}{3}\right)$ on a $128^{3}$ grid.

We investigate (isothermal) triphase separation in an ideally perfectly symmetric and highly viscous (zero fluidity) ternary mixture, which is instantaneously quenched from a stable state having the initial composition $x_{A}=\left(\frac{1}{3},\frac{1}{3},\frac{1}{3}\right)$ in the one-phase region to an unstable state (at a smaller temperature) corresponding to point A in the phase diagram in Figure 1. Assuming an instantaneous quench to a uniform temperature, the initial field for each mass fraction is specified as random (delocalized) concentration fluctuations superimposed on a uniform $x_{i,0}=\frac{1}{3}$ composition. From this simulation (denoted as case I, more precisely defined below), we show isosurfaces of phase η vs. those for phase ξ at one particular instant in time in Figure 2, suggesting that phase separation for component 3 is faster than that for component 1. For a more quantitative characterization of the the phase-separation kinetics in such a highly viscous system, we looked at the temporal evolution of three characteristic length scales of single-phase microdomains, defined as

(9) $$L_{i}\left(t\right)=\frac{1}{x_{i,rms}^{2}}\sum_{k}\frac{|{\hat{x}}_{i}{\left(k\right)|}^{2}}{\left|k\right|}\;\left(i=1,\;2,\;3\right),$$

with ${\hat{x}}_{i}\left(k\right)$ denoting the Fourier coefficient for the *i*th mass fraction at wavevector k. In other words, $L_{i}$ is the same as the the integral scale of the pair-correlation function associated with the *i*th mass fraction. As can be seen (Figure 3), at first the integral scales for different components are essentially coincident as they undergo a rapid transient with an undershoot behavior that delays phase separation, after which the three phases seem to grow together, with a coarsening rate that could be expressed approximately as $L_{i}∼\tau^{0.07}$ (where $\tau \equiv 10^{-5}t$); note, however, that there does not seem to be enough of a scaling range for this power law to be physically meaningful. Finally, at about $\tau \approx 4\times 10^{-4},$ a symmetry-breaking event occurs as component 3 continues to separate quickly, while phase separation for the other two components seems to be delayed. From our simulation results, this behavior seems to be robust, in that very similar temporal histories are obtained for the reciprocal first and second moments of the structure factor associated with the *i*th mass fraction, i.e.,

(10) $$\lambda_{i}^{\left(1\right)}=\frac{\sum_{k}{\left|{\hat{x}}_{i}\left(k\right)\right|}^{2}}{\sum_{k}|{\hat{x}}_{i}{\left(k\right)|}^{2}\left|k\right|},\;\mathrm{and}\;\lambda_{i}^{\left(2\right)}={\left[\frac{\sum_{k}{\left|{\hat{x}}_{i}\left(k\right)\right|}^{2}}{\sum_{k}|{\hat{x}}_{i}{\left(k\right)|}^{2}{\left|k\right|}^{2}}\right]}^{1/2}.$$

We also looked at the temporal evolution of the separation depth for each component, defined as

(11) $$s_{i}=\langle \frac{x_{i}\left(r\right)-x_{i,0}}{x_{i,eq}\left(r\right)-x_{i,0}}\rangle ,$$

where

(12) $$x_{i,eq}\left(r\right)=\left\{\begin{array}{cc} 1-2x^{*} & \mathrm{if}\;x_{i}\left(r\right)>x_{i,0},\\ x^{*} & \mathrm{if}\;x_{i}\left(r\right)<x_{i,0}. \end{array}\right.$$

**Figure 4:**
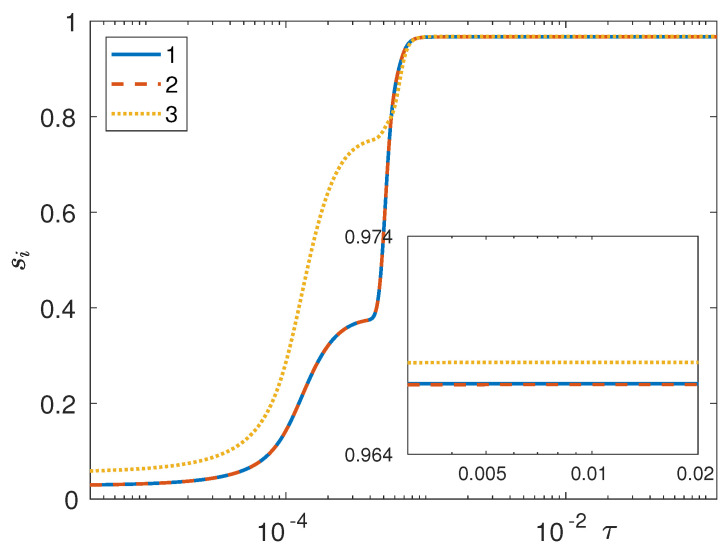
Separation depths vs. time (diffusive time units) from 3D phase-field simulation (case I) of a perfectly symmetric ternary mixture with Ψ=4 and global composition $x_{A}=\left(\frac{1}{3},\frac{1}{3},\frac{1}{3}\right)$ on a $128^{3}$ grid with $s_{1}\left(0\right)=s_{2}\left(0\right)$ and $s_{3}\left(0\right)\approx s_{1}\left(0\right)+s_{2}\left(0\right)$.

Clearly, this definition is the same as that employed for studying phase separation in a binary system [38,39,41,49,52,54,55,56]; in fact, the reason that the same definition can be brought to bear on a ternary system is that, based on the mixture phase diagram in Figure 1, both phases in any one pair of coexisting phases at equilibrium possess the same $x_{i,eq}$ (with *i* denoting the component at the opposite vertex in the phase diagram). As can be seen (Figure 4), phase separation for component 3 is faster than that for the remaining two components, thus confirming the (qualitative) conclusions that were reached by observing Figure 2. In Figure 4 we can see that $s_{3}$ at any instant in time is larger than $s_{1}$ and $s_{2}$, but for a very small subrange of simulated times wherein all three separation depths show a very rapid increase to their steady state values. In fact, in this case (I) the initial fields for the first two components were taken to be numerically identical in order to have $s_{1}=s_{2}$ at the initial time; consequently, conservation of mass necessarily implies $s_{3}\left(0\right)>s_{1}\left(0\right)$ [in fact, it is easily seen that $s_{3}\left(0\right)\approx 2s_{1}\left(0\right)$].

**Figure 5:**
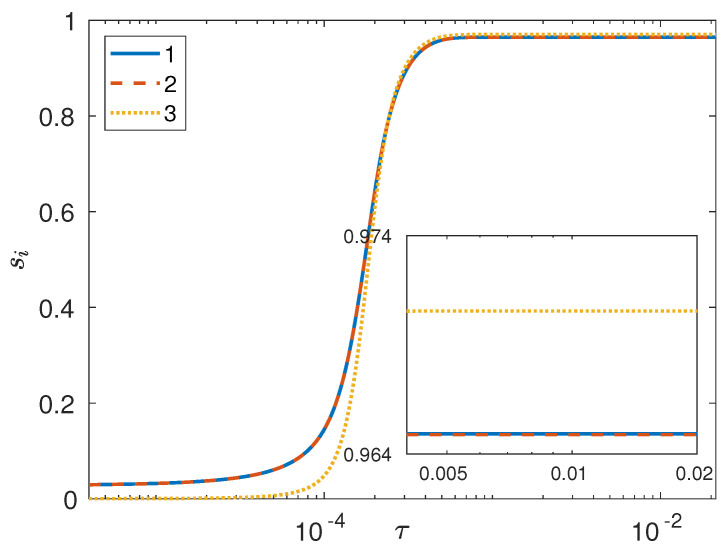
Separation depths vs. time (diffusive time units) from 3D phase-field simulation (case II) of a perfectly symmetric ternary mixture with Ψ=4 and global composition $x_{A}=\left(\frac{1}{3},\frac{1}{3},\frac{1}{3}\right)$ on a $128^{3}$ grid with $s_{1}\left(0\right)=s_{2}\left(0\right)$ and $s_{3}\left(0\right)=0$.

**Figure 6:**
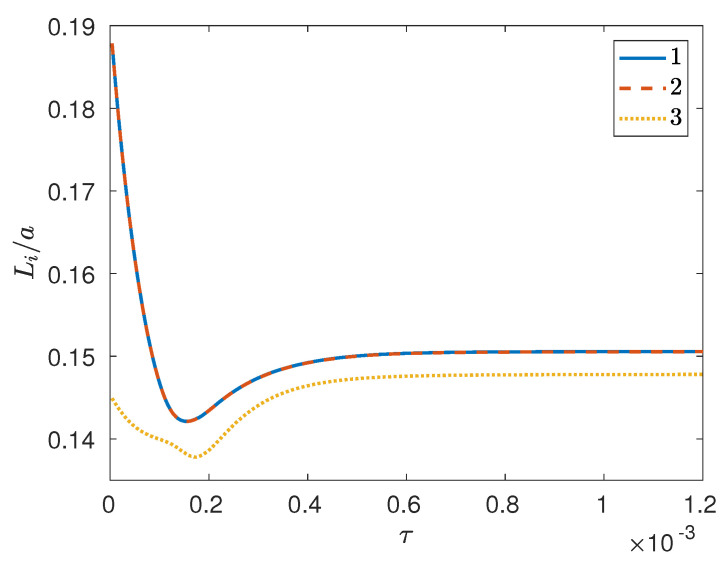
Integral scales vs. time (diffusive time units) from 3D phase separation simulation (case II) of a perfectly symmetric ternary mixture with Ψ=4 and global composition $x_{A}=\left(\frac{1}{3},\frac{1}{3},\frac{1}{3}\right)$ on a $128^{3}$ grid with $s_{1}\left(0\right)=s_{2}\left(0\right)$ and $s_{3}\left(0\right)=0$.

In another simulation (denoted as case II below), the initial conditions were generated such that the random noise for component 2 exactly canceled that for 1 at the initial time; this necessarily implies $s_{3}\left(0\right)=0$, along with $s_{1}\left(0\right)=s_{2}\left(0\right)$. In this case (see Figure 5), phase separation for component 3 is slower than that for the remaining two components, given that the separation depth for component 3 remains smaller (and in fact close to zero) than that for 1 and 2 for some time after the initial time, after which all separation depths show a very rapid increase to their steady state values. Once again, even though $s_{1}$ and $s_{2}$ remain identical for all time, the steady state value for 3 turns out to be slightly larger than that for the first two components. Also note that in this simulation the temporal evolution of the integral scales is very different from that corresponding to case I; in fact, even though the integral scales for 1 and 2 are essentially identical for all time (see Figure 6), the integral scale for 3 is systematically smaller than that for 1 (or 2) while showing a similar decrease–increase behavior. Once again, this temporal evolution seems to be robust, in that very similar temporal histories are also displayed by the reciprocal first and second moments of the structure factor.

Summarizing, in simulation case I [such that $s_{3}\left(0\right)>s_{1}\left(0\right)=s_{2}\left(0\right)$] based on the temporal evolution of the integral scales, it seemed as though a symmetry-breaking event took place after which the integral scale vs. time dependences showed a different phase-separating behavior for components 1 and 2 as opposed to 3. However, looking at the separation depth in this case reveals that phase separation for component 3 is always faster than that for the first two components, implying that there can be no symmetry among the three components at any instant in time. A similar conclusion is arrived at by looking at simulation case II [such that $0=s_{3}\left(0\right)<s_{1}\left(0\right)=s_{2}\left(0\right)$], wherein phase separation for component 3 was always slower than that for the remaining components. Furthermore, in this case the integral scale for 3 was always less than that for 1 (or 2), providing even stronger evidence against any presumption of symmetry among the three components during a triphase segregation process.

As a final note, it should be acknowledged that, even though a perfectly symmetric ternary mixture does not exist in practice, our phase-field ternary mixture model has been successfully employed to simulate the so-called continuous-phase/dispersed-circular/dispersed-circular (a.k.a. dual discrete particle) phase morphology for a phase-separating symmetric polymer-polymer-polymer system [57]. Although improved agreement between model predictions and experimental results of phase morphology for a polystyrene/PMMA/polybutadiene blend was obtained in simulations that allowed for different diffusivities and Flory parameters [58], simulation results that instead assumed equality between all diffusivities, Flory parameters, and chain lengths showed that a phase-field ternary mixture model [59] is able to capture the main features of the previously noted phase morphology [58,60].

## 4. Conclusions

We have presented 3D numerical results of an isothermal phase-field ternary mixture model in application to a symmetric and highly viscous (zero fluidity parameter) three-component liquid mixture as it separates into three phases after a thermal quench from a stable state in the one-phase region. In particular, we have shown the temporal evolution of basic segregation statistics such as the integral scale of the pair-correlation function and the separation depth for each component. In fact, for a ternary system the latter quantity can be introduced using the same definition of separation depth as employed for measuring the phase separation rate in a binary system. In addition, we have shown that the triphase segregation process considered herein is inherently asymmetrical since it can occur in either one of two ways: depending on the details of the noise used for building the initial conditions, phase separation for the third component will be faster or slower than that for the remaining components.

## Abbreviations

The following abbreviations are used in this manuscript:

2D	Two-Dimensional
3D	Three-Dimensional
NRTL	Non-Random, Two-Liquid
